# Effects of aging on the biomechanical properties of the lung extracellular matrix: dependence on tissular stretch

**DOI:** 10.3389/fcell.2024.1381470

**Published:** 2024-04-05

**Authors:** Anna Ulldemolins, Maria Narciso, Héctor Sanz-Fraile, Jorge Otero, Ramon Farré, Núria Gavara, Isaac Almendros

**Affiliations:** ^1^ Unitat de Biofísica i Bioenginyeria, Facultat de Medicina i Ciències de la Salut, Universitat de Barcelona, Barcelona, Spain; ^2^ The Institute for Bioengineering of Catalonia (IBEC), The Barcelona Institute of Science and Technology, Barcelona, Spain; ^3^ CIBER de Enfermedades Respiratorias, Madrid, Spain; ^4^ Institut d’Investigacions Biomèdiques August Pi i Sunyer, Barcelona, Spain

**Keywords:** lung extracellular matrix, aging, lung volume, biomechanical properties, mesenchymal stromal cells

## Abstract

**Introduction:** Aging induces functional and structural changes in the lung, characterized by a decline in elasticity and diminished pulmonary remodeling and regenerative capacity. Emerging evidence suggests that most biomechanical alterations in the lung result from changes in the composition of the lung extracellular matrix (ECM), potentially modulating the behavior of pulmonary cells and increasing the susceptibility to chronic lung diseases. Therefore, it is crucial to investigate the mechanical properties of the aged lung. This study aims to assess the mechanical alterations in the lung ECM due to aging at both residual (RV) and functional (FV) lung volumes and to evaluate their effects on the survival and proliferation of mesenchymal stromal cells (MSCs).

**Methods:** The lungs from young (4-6-month-old) and aged (20-24-month-old) mice were inflated with optimal cutting temperature compound to reach FV or non-inflated (RV). ECM proteins laminin, collagen I and fibronectin were quantified by immunofluorescence and the mechanical properties of the decellularized lung sections were assessed using atomic force microscopy. To investigate whether changes in ECM composition by aging and/or mechanical properties at RV and FV volumes affects MSCs, their viability and proliferation were evaluated after 72 h.

**Results:** Laminin presence was significantly reduced in aged mice compared to young mice, while fibronectin and collagen I were significantly increased in aged mice. In RV conditions, the acellular lungs from aged mice were significantly softer than from young mice. By contrast, in FV conditions, the aged lung ECM becomes stiffer than that of in young mice, revealing that strain hardening significantly depends on aging. Results after MSCs recellularization showed similar viability and proliferation rate in all conditions.

**Discussion:** This data strongly suggests that biomechanical measurements, especially in aging models, should be carried out in physiomimetic conditions rather than following the conventional non-inflated lung (RV) approach. The use of decellularized lung scaffolds from aged and/or other lung disease murine/human models at physiomimetic conditions will help to better understand the potential role of mechanotransduction on the susceptibility and progression of chronic lung diseases, lung regeneration and cancer.

## 1 Introduction

During aging, lungs undergo structural and functional changes, including loss of elasticity and reduced pulmonary remodeling and regenerative capacity. These changes culminate in a decline in lung function and an increased susceptibility to chronic lung diseases (Cho and Stout-Delgado, 2020). There is growing evidence that most biomechanical changes are mediated by alterations in lung extracellular matrix (ECM) composition, as observed in chronic obstructive pulmonary disease (COPD), idiopathic pulmonary fibrosis (IPF), and aging (Burgstaller et al., 2017; Suki et al., 2022a; Burgess and Harmsen, 2022; Hoffman et al., 2023; Júnior et al., 2023; Mebratu et al., 2023). In fact, these alterations can lead to changes in ECM stiffness, and through mechanotransduction, they could modulate the behavior of pulmonary cells, facilitating disease progression. However, there is a scarcity of studies directly examining how age-related lung ECM alterations could modify ECM stiffness and, consequently, cellular responses (Faner et al., 2012).

The pulmonary connective tissue of the lung is composed of cells and the ECM, which is composed of a variety of biological macromolecules that are differently organized according to tissue or organ. Indeed, the mechanical properties of the ECM are determined by its composition, with collagen, elastin, and proteoglycans playing critical roles in the lung (Suki and Bates, 2008). Golding et al. postulated that the lungs gradually become more rigid with age due to altered expression of ECM proteins, including laminin, elastin, and fibronectin (Godin et al., 2016). Lung decellularization is a well-established technique for understanding the role of the composition and structural changes experienced by the ECM in different lung diseases and aging (Uriarte et al., 2018). Moreover, decellularized lungs can also be used as scaffolds for cell culture to better investigate specific ECM-cell interactions more effectively. The ECM protein content alterations induced by aging could lead to mechanical changes in the lung. In this regard, Melo et al. reported a tendency of increase in local stiffness measured by atomic force microscopy (AFM) in functional volume (FV) inflated decellularized lungs from young and aged mice (Melo et al., 2014). However, by using non-inflated lungs, other studies have observed significant age-related increases in stiffness in parenchymal and vessel compartments (Sicard et al., 2018) and even, a decrease (Miura, 2022). Therefore, the scarce available data are inconclusive.

The volume of gas in the intrathoracic airways is determined by the properties of the lung parenchyma, surface tension, and the forces exerted by respiratory muscles. In physiological conditions, because of the complex structure of the lung and its cyclic deformation during spontaneous breathing, pulmonary cells are continuously exposed to different levels of mechanical stresses. Interestingly, stretch can induce strain-softening of acellular fibrotic lungs at the nanoscale, as recently reported (Júnior et al., 2021), revealing the importance of conducting these measurements in physiomimetic conditions. Conversely, most available studies aiming to understand the biomechanical properties of the aged lung were done at residual volume (RV) (Sicard et al., 2018; Miura, 2022), since the lungs collapse after their removal. In fact, if mechanical measurements using residual (non-inflated) or functional volumes (Hopkins and Sharma, 2022) lead to consistent results in aging studies, is still unknown.

Here, we aimed to determine the biomechanical changes induced by aging in acellular lungs at RV and at physiologically-inflated conditions or functional volume (FV). The mechanical measurements were carried out at the nanoscale using AFM. To this end, an *in situ* decellularization method allowed us to decellularize the ECM for subsequent analysis of its mechanics and composition. Finally, the acellular lung scaffolds were repopulated with mesenchymal stromal cells (MSCs) to investigate whether their viability and proliferation are affected by aging and/or tissular stretch.

## 2 Materials and methods

### 2.1 Sample collection

**FIGURE 1:**
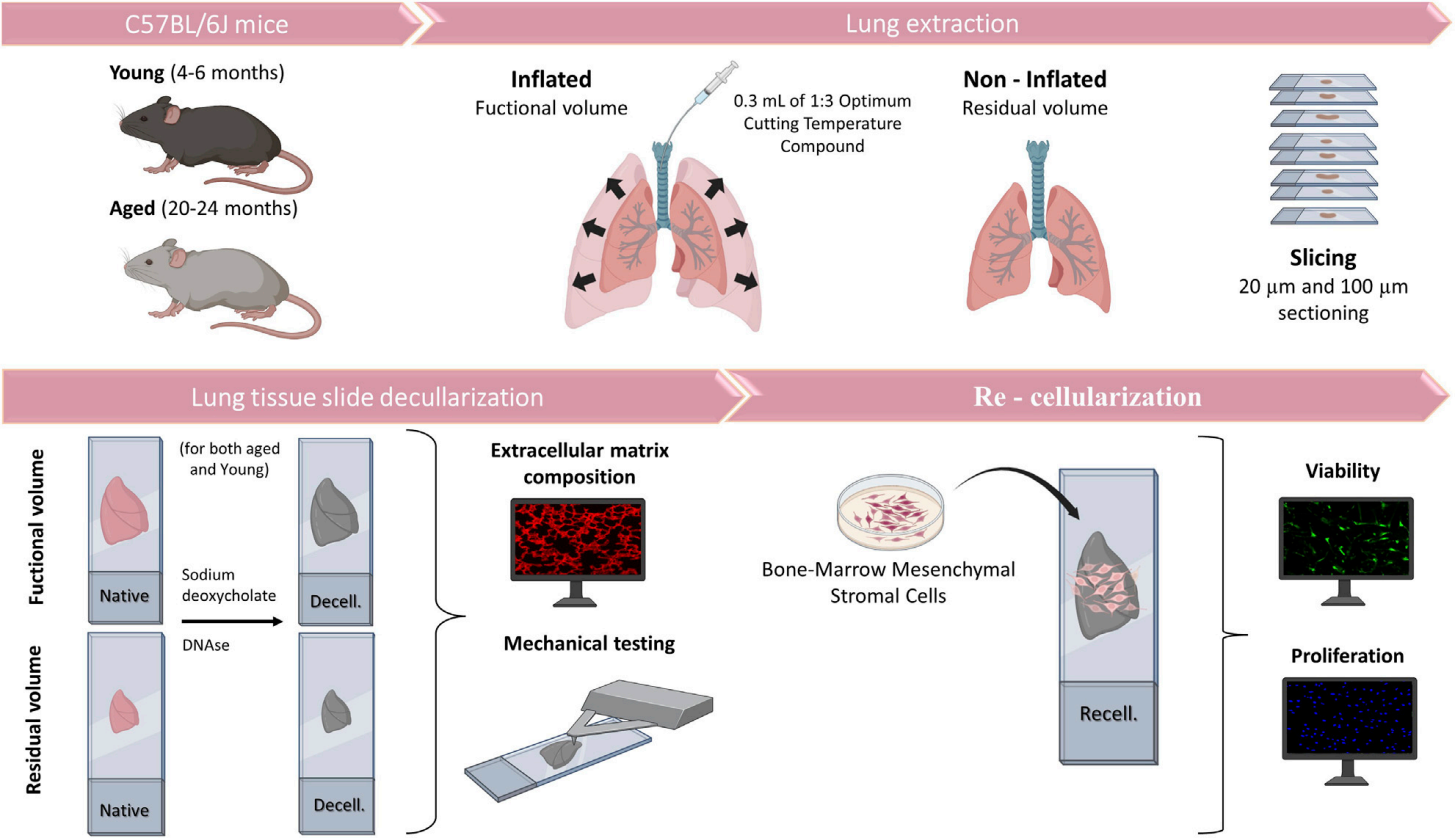
Schematic representation of the methodology.

This work was approved by the Ethical Committee of the University of Barcelona and Generalitat de Catalunya (OB 168/19 and 10972). Lungs were harvested *en bloc* with the heart from eight young (4-6-month-old) and eight aged (20–24-month-old) male C57BL/6J mice (Charles River Laboratories, Saint Germain sur L’arbresle, France). The lungs were subsequently inflated with 1:3 dilution of optimal cutting temperature (OCT, Tissue-Tek, Sakura, Torrance, CA, United States) compound with 0.3 mL to reach FV (Mitzner et al., 2001; Uriarte et al., 2016). Another group without inflation, at RV was sampled in parallel (Figure 1). Mice lungs were sliced at 20 and 100 µm using a cryostat with a −24°C temperature setting. The sections were placed in a positively charged glass slide (Superfrost Plus, Thermo Fisher Scientific, Waltham, MA, United States) and allowed to air-dry 15 min before being stored at −80°C until needed.

### 2.2 Lung decellularization

The lung slices were decellularized by using SDS followed by DNase leading to minimal alterations to ECM composition and mechanics in comparison with other methods (Narciso et al., 2021; Narciso et al., 2022a; Narciso et al., 2022b). In brief, acellular sections were created through a series of sequential washes while the lung sections remained attached to the glass slides, allowing us to maintain the physiological stretch (FV) at functional residual capacity (FRC) +1/2 tidal volume (Rojas-Ruiz et al., 2023) or non-inflated (RV). Before the decellularization process, the tissue sections were allowed to thaw at room temperature (RT) for 20 min. The OCT was removed by immersing the sections in PBS for 20 min. The cells were lysed through two successive 10-min rinses with ultrapure water, followed by two 15-min incubations with 2% sodium deoxycholate (SD). After removing SD with three PBS washes, a 20-min incubation in a DNAse I solution (0.3 mg/mL, 5 mM MgCl_2_, 5 mM CaCl_2_ in 1 mM Tris-HCl) was carried out and then it was removed through three consecutive 5-min PBS washes. Decellularization was assessed by DNA immunostaining (Hoechst, Thermo Fisher Scientific).

### 2.3 Atomic force microscopy measurements and data analysis

The lung ECM from eight young (n = 4 RV/FV conditions) and 16 aged animals (n = 8 RV/FV conditions) was mechanically probed in 20 µm-thick scaffolds immediately after decellularization by using cantilevers with a nominal spring constant (k) of 0.03 N/m and featuring a silicon oxide bead (5 µm in diameter) attached to their end (Novascan Technologies, IA) (Narciso et al., 2022a; Narciso et al., 2022c).

For each measurement point, the sample underwent five indentations to minimize measurement-to-measurement variability using 15 µ/s ramping speeds. Within each region, measurement points were separated by a minimum of 20 µm. We measured 3-5 points in each region and performed measurements in 3-5 different regions within the sample.

Considering that a spherical tip was used in this study, we deemed the Hertz contact model for a sphere indenting a semi-infinite half-space as the most suitable approach, in alignment with prior research in the field (Liu et al., 2010; Jorba et al., 2019; Júnior et al., 2021).

To determine the model’s parameters, we employed a custom MATLAB code to fit each force-deflection curve (Jorba et al., 2019). Cellular viscosity was determined from force–distance curves using the method described by (Rebelo et al., 2014). Unspecific adhesion force was estimated as the most negative force value within the withdrawal part of the force-indentation curve.

### 2.4 Extracellular matrix immunofluorescence assay and data analysis

Both decellularized and native non-inflated (RV) lungs ∼20 µm-thick sections (n = 3) were stained for laminin, fibronectin, collagen I to characterize the matrix proteins. For native sections, immunofluorescence staining was performed after consecutive washes with PBS to remove the OCT. For decellularized sections, the staining protocol was performed immediately after the decellularization procedure. The tissue was fixed using 4% paraformaldehyde for 10 min at room temperature. Samples were then blocked using a buffer composed of 10% fetal bovine serum (FBS) and supplemented with 3% bovine serum albumin (BSA) for 1 h at RT. Primary antibodies against fibronectin (1:100, rabbit anti-fibronectin, ab2413, Abcam), laminin (1:100, rabbit anti-laminin, Thermo Fisher Scientific) and type I collagen (rabbit anti-collagen type I, Abcam, 1:100) were incubated overnight at 4°C and constant agitation (80 rpm). Sections were then rinsed three times with the blocking buffer. The secondary antibody (goat anti-rabbit Cy3, Thermo Fisher Scientific, 1:200) was incubated for 2 h, at 37°C and constant agitation (80 rpm). Three 15-min rinses with PBS were performed. DNA of cellular and acellular samples was stained by incubation with Hoechst 33342 (Thermo Fisher Scientific) for 20 min at 80 rpm in an orbital shaker followed by three 5-min PBS washes. Finally, samples were mounted in Fluoromount mounting media (Thermo Fisher Scientific) and stored at 4°C. Epifluorescent images were acquired with a Leica SP5 inverted microscope equipped with a CCD camera (C9100, Hamamatsu Photonics K.K. Hamamatsu, Japan) and using a ×10 and ×20 Plan Fluor objective (Nikon).

Multi-channel TIFF images of the ECM stained with fibronectin, laminin and type I collagen were analyzed. The images were subjected to kernel density estimation using a custom MATLAB (MATLAB, The MathWorks Inc., MA, United States) script to evaluate the pixel intensity distribution across the image. Briefly, a lower and upper threshold for pixel intensity was applied to isolate the regions of interest. Both weak and intense light areas were quantified by calculating the area under the intensity distribution curve before and after the peak intensity. The results were normalized by the total area to account for variations in ECM deposition across samples. Comparisons between both groups were made based on the distribution of pixel intensities. The percentage of activated pixels was computed for each image.

### 2.5 Mesenchymal stromal cells repopulation, cell viability and proliferation assays

Primary human Bone Marrow-Derived MSCs (PCS-500–012, ATCC) were cultured in MSCs Basal Medium (PCS-500–030, ATCC) following manufacturer’s instructions at 37°C, 5% CO_2_ and 95% relative humidity.

Lungs from 20 animals (n = 5, young/aged and RV/FV) were decellularized. Then, ∼100 µm-thick scaffolds were washed with PBS and 5·10^4^ cells/cm^2^ MSCs were seeded on top of the lung scaffolds. Control cultures were seeded on traditional culture plates (TCP). After 72 h, samples were stained using the LIVE/DEAD Viability/Cytotoxicity kit (L-3224, Invitrogen) (Bonenfant et al., 2013; ONeill et al., 2013; Syed et al., 2014). F-Actin (phalloidin, Thermo Fisher Scientific) and Ki67 (Thermo Fisher Scientific) were stained and visualized by a Nikon D-Eclipse Ci confocal microscope with a ×20 Plan Apo immersion oil objective (Nikon). Image quantification was performed using a custom MATLAB script. Initially, images in both Hoechst and Ki67 channels were converted to grayscale. User-defined thresholds were applied to distinguish cellular regions based on pixel intensity. Following the thresholding, region properties such as area, major axis length, and centroid were computed for individual cellular regions. To exclude artifacts and non-cellular elements, a minimum diameter filter was applied, removing any identified regions smaller than a pre-defined pixel size. To compensate for potential positional differences between the Hoechst and Ki67 staining, a dilation operation was performed on the Hoechst image. Ki67-positive cells were identified by overlaying the dilated Hoechst image with the Ki67 image, ensuring colocalization. Finally, the percentage of Ki67-positive nuclei relative to the total Hoechst-stained nuclei was computed to determine cellular proliferation.

### 2.6 Statistical analysis

All values are expressed as mean ± standard error (SE). Comparison in the expression of ECM protein content between young and aged lung samples was carried out with a *t*-test. Two-way ANOVA was used to compare changes in biomechanical properties and MSCs viability and proliferation as induced by age (young/aged) and/or tissular stretch (RV vs. FV). Student t-tests were employed to ascertain differences among groups. Statistical significance was considered when *p* < 0.05.

## 3 Results

### 3.1 Aging modifies the composition of the extracellular matrix

**FIGURE 2:**
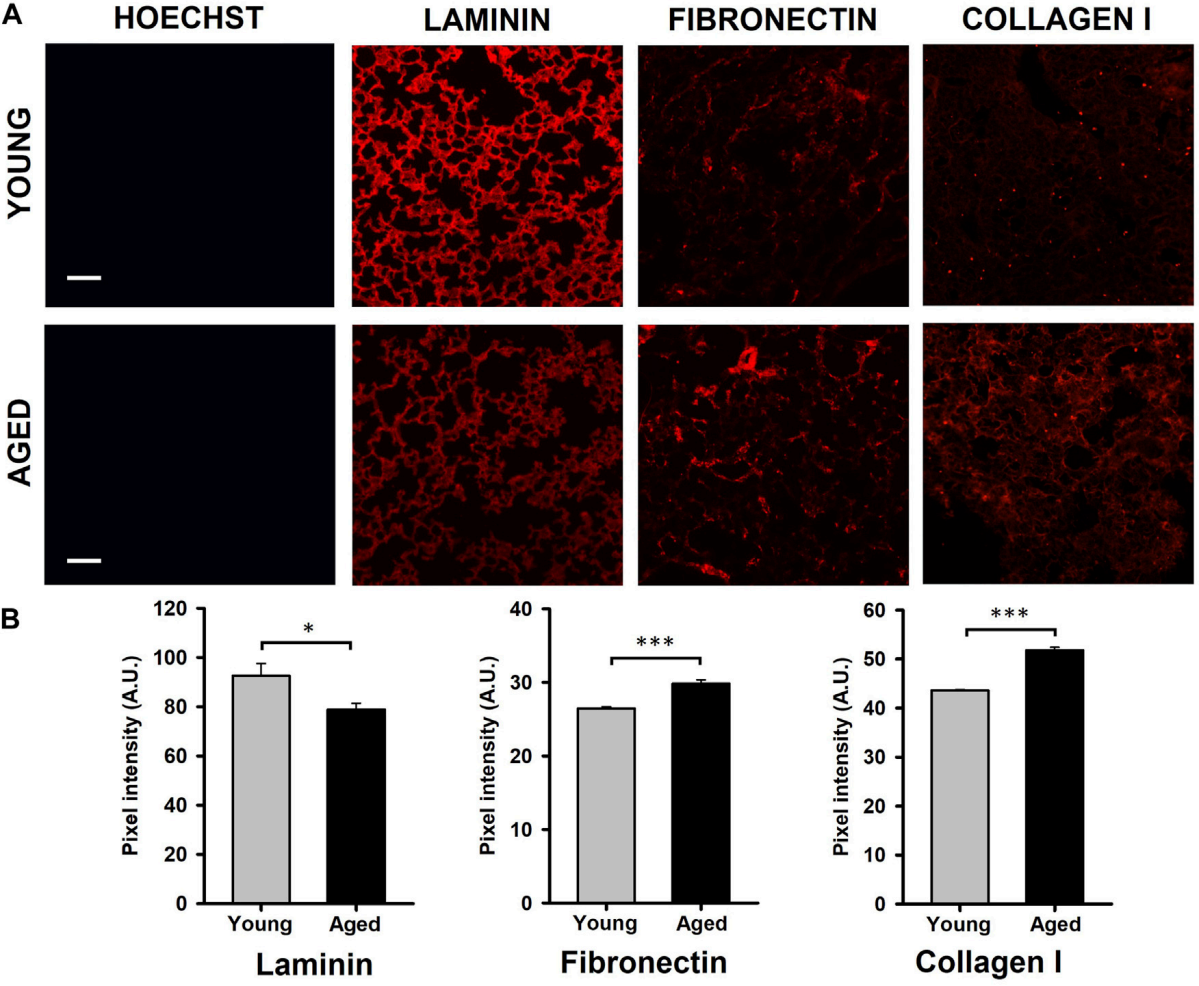
Quantification of ECM protein content. **(A)** Illustrative images of decellularized lung ECM stained for laminin, fibronectin, and collagen type I from young and aged mice. **(B)** Aging promotes a reduction of laminin and increases of fibronectin and collagen type I. Scale bar = 100 μm, values are represented as mean ± SE, *p < 0.05, **p < 0.01 and ***p < 0.001.

Results obtained by the quantification of immunofluorescence images showed that the protein composition of the lung ECM is affected by aging. Specifically, a significantly decreased level of laminin (∼15%, *p* = 0.041) and increased expressions of fibronectin (∼15%, *p* < 0.001) and collagen (19%, *p* < 0.001) were observed in samples from aged animals (Figures 2A, B).

### 3.2 Extracellular matrix biomechanics depends on aging and tissue stretch

**FIGURE 3:**
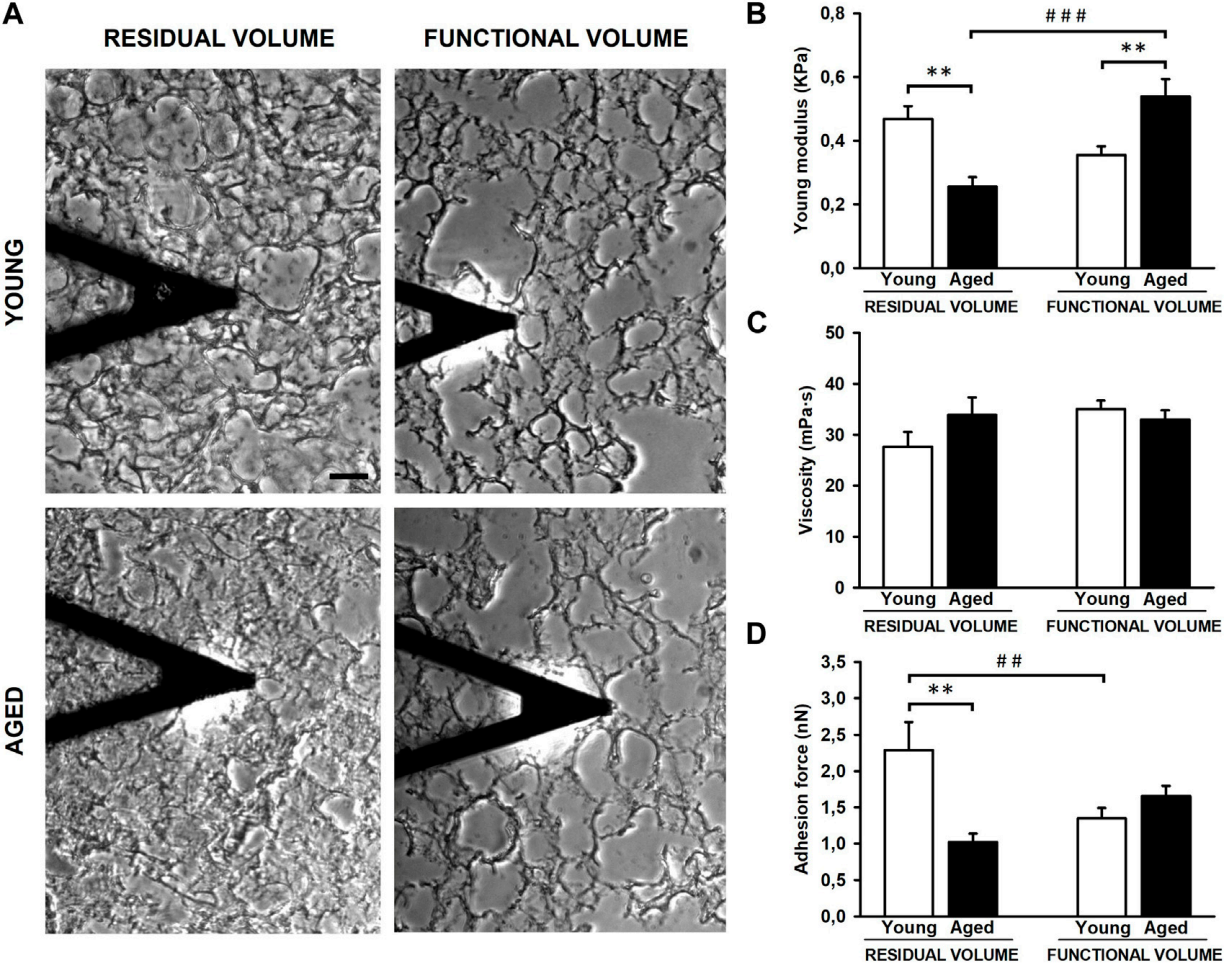
Mechanical properties of decellularized lung extracellular matrix (ECM). Atomic force microscopy images of lung samples from young and aged mice in residual (RV) and functional (FV) volumes **(A)**. ECM stiffness of aged animals is softer compared to youngers at RV but stiffer at FV **(B)**. No differences were observed in viscoelasticity **(C)** and unspecific adhesion forces are higher in young ECM only at RV **(D)**. Scale bar = 50 μm, values are represented as mean ± SE, Comparisons between young/aged: **p < 0.01 and between RV/FV: ^##^ *p* < 0.01 and ^###^ *p* < 0.001.

The experimental setting allowed us to measure the lung ECM biomechanics by AFM at RV (collapsed) and at FV (stretched) conditions (Figure 3A). Regarding lung ECM stiffness, the Young’s modulus, measured at RV, was significantly lower (*p* = 0.003) in aged mice (0.26 ± 0.03 kPa) when compared to that assessed in younger animals (0.47 ± 0.04 kPa) (Figure 3B). Conversely to RV, the stiffness of the lung ECM, measured at FV, was significantly increased (*p* = 0.002) in aged mice (0.54 ± 0.06 kPa) with respect to the younger counterparts (0.36 ± 0.03 kPa). Thus, these findings reveal that the effects of aging on ECM stiffness strongly depend on lung volume (*p* < 0.001). In this context, when comparing RV and FV measurements, it is noteworthy that aged lungs manifest a significant ∼ 2-fold increase in ECM stiffness under FV conditions (*p* < 0.001). Conversely, a noticeable trend towards a decrease (*p* = 0.063) is observed in their younger counterparts (Figure 3B). Regarding ECM viscosity, no changes were observed between young/aged and RV/FV conditions (Figure 3C).

The AFM measurements also allowed us to measure the unspecific adhesion forces exerted by the ECM. The adhesion forces were similar between young (1.35 ± 0.15 nN) and aged (1.65 ± 0.14 nN) lung acellular ECM at FV conditions. However, the adhesion measured in young lung ECM (2.29 ± 0.39 nN) was ∼2-fold higher (*p* = 0.001) with respect to aged counterparts (1.02 ± 0.12 nN) at RV conditions (Figure 3D). In a similar fashion to ECM stiffness measurements, the lung ECM exerts different adhesion forces behavior between both lung volumes (RV and FV) (*p* = 0.006) only in young animals.

### 3.3 MSCs viability and proliferation on decellularized lung slices do not depend on the age and stretch of the lung

**FIGURE 4:**
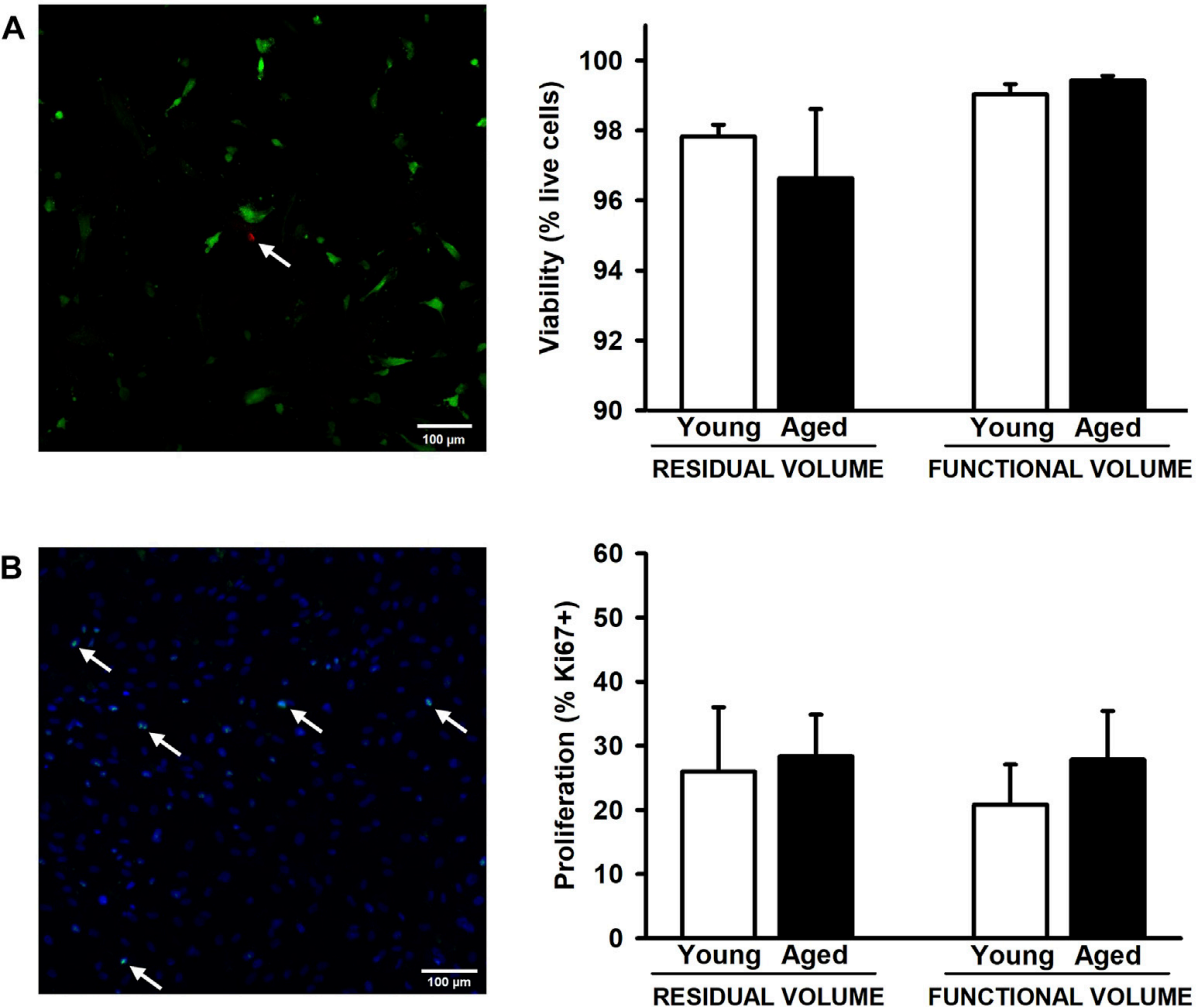
Proliferation and viability of mesenchymal stromal cells cultured on decellularized lung ECM. No changes were observed in viability **(A)** and proliferation **(B)** among all experimental groups. Arrows indicates some dead cells **(A)** and Ki67+ cells **(B)**. Scale bar = 100 μm, values are represented as mean ± SE.

After 72 h of the lung scaffold repopulation with MSCs, they show a spread morphology indicative of their attachment to the scaffold. Additionally, no effects due to aging and lung volume were observed in cell viability presenting values higher than 96.5% in all groups (Figure 4A). In addition, the proliferation of MSCs, measured as a percentage of positive Ki67 cells, did not present significative differences in any condition (Figure 4B).

## 4 Discussion

This study provides novel insights into how age-induced alterations in the ECM composition are translated into biomechanical changes. Of particular interest is the discovery that variations in ECM stiffness and the adhesion forces exerted by aging are notably influenced by whether the lung is in a non-inflated or stretched state at RV and FV conditions, respectively. The model presented could provide novel insights in regenerative medicine, cancer, and other pulmonary chronic diseases during aging.

From rodent models, the studies that compared elderly to younger animals found a different biological response (Bos et al., 2018). In this regard, it is well established that there is a general pro-fibrotic response with aging in several organs and tissues, with particular relevance in the lungs. Specifically, it has been described that age-induced lung fibrosis is promoted by several factors, including genomic instability, telomere attrition, epigenetic alterations, mitochondrial dysfunction, cellular senescence, and stem cell exhaustion (Thannickal, 2013). For instance, decellularized lungs from aged rodents were reconstituted with bronchial epithelial cells and lung fibroblasts, exhibiting an altered gene expression of ECM proteins compared to young lungs (Godin et al., 2016). Even though ECM protein composition has been extensively studied in healthy lungs and in certain pulmonary diseases, the available data focused on ECM changes during aging is still very scarce. Collagens and fibronectin are considered two of the main ECM components and their dysregulation has been associated with the pathogenesis and progression of several chronic lung diseases, which predominantly occur in the elderly (Onursal et al., 2021). However, it is unknown whether ECM alterations induced by aging could lead to a predisposition to the development of such diseases. In this context, it has been reported that the synthesis and maturation of different types of collagens could be influenced by aging, resulting in distinct changes in collagen composition and in the collagen crosslinks (Sokocevica et al., 2011; Onursal et al., 2021). Interestingly, human bronchial epithelial cells and lung fibroblasts cultured on decellularized aged lung scaffolds showed a decreased expression of laminins α3 and α4, elastin and fibronectin, and elevated collagen, compared to young lungs (Godin et al., 2016). In contrast, Takubo Y. et al. reported increased total collagen content in lung ECM from 20-month-old mice, but no changes in collagen proportion and elastin content were found (Takubo et al., 1999). Furthermore, the degradation of collagen is also an essential process to resolve lung fibrosis. Podolsky MJ. et al. have shown that cell-mediated collagen uptake and degradation are diminished in the lungs of 10-month-old mice, suggesting that this impairment could contribute to age-related fibrosis (Podolsky et al., 2020). These results are also according to the findings observed by Huang K et al. showing a reduction of elastin fibers and increased density of collagen leading to an increased lung compliance during aging (Huang et al., 2007a; Huang et al., 2007b). The present study is focused on collagen I, laminin and fibronectin, two ECM components that contribute to ECM biomechanics of the static lung (Álvarez et al., 2015). As expected, all ECM components assessed in decellularized lung scaffolds were altered in a different manner by aging (Figure 2). According to previous results, collagen I and fibronectin were increased, and laminin expression was reduced in 2-year-old mice with respect to younger animals. Therefore, our results and prior data collectively suggest that aged lungs display unique ECM components, and those alterations could contribute to the onset of age-related lung diseases.

It may be expected that changes that occur in the composition of the ECM during aging may have an impact on its biomechanical properties. This information is crucial to better understand the physiology and pathophysiology of the lungs, considering that cell-ECM interaction has been proposed to modify numerous cellular responses during development, aging and disease (Bonnans et al., 2014; Mebratu et al., 2023). In this context, numerous works have estimated the mechanical properties of murine and human lungs at macroscale by indenting lung biopsies, stretching lung tissue strips, measuring whole lung compliance and elastance, and bulk modulus (Lai-Fook and Hyatt, 1985; Suki et al., 2022b). Although there are discrepancies on the Young’s modulus values, all studies suggest an increased stiffness in the aged lung (Suki et al., 2022b). However, very limited data is currently available in the literature at nanoscale level. In accordance to our results, Melo et al. reported a tendence of increase on local stiffness in decellularized murine lungs at FV conditions (Melo et al., 2014). Sicard D et al. observed that non-decellularized lung tissues from 41 to 60 years/o subjects were stiffer than lung samples from 11 to 30 years/o subjects by using AFM in 10-µm thickness tissue slices from organ donors (Sicard et al., 2018). In contrast, Miura K suggests that lungs are gradually softening with age. The lung tissue stiffness from human samples was correlated to the speed of sound measured using a scanning acoustic microscope (Miura, 2022). Hence, the scarce existing data on lung biomechanics and aging appears to be contentious, likely due to variations in species, methodologies, and the use of native or decellularized samples across different studies. Another notable common characteristic in biomechanical studies of the lung is the absence of agreement regarding lung tissue stretch during measurements. Indeed, most studies relying on *ex vivo* experimental assessments use non-inflated lungs at RV (Sicard et al., 2018; Miura, 2022). However, recent observations highlight that alterations in tissue stretch during breathing can correlate with changes in ECM stiffness (Jorba et al., 2019). Given the variations in ECM content and the modifications in protein ECM crosslinks associated with certain lung diseases and aging, it is imperative to consider the level of tissue stretch during AFM measurements. In this manuscript, we describe for the first time how aging modifies some biomechanical properties of the lung at the microscale in non-inflated (RV) and physiologically inflated (FV) conditions. To reach the FV, the lungs were inflated with 0.3 mL of OCT solution (1:3 diluted) as previously reported (Mitzner et al., 2001; Uriarte et al., 2016). The amount instilled was the same in both groups considering the minimal change in lung volume (∼20%) between young (4–6 months old) and aged (20–24 months old) mice (Elliott et al., 2016). As expected, changes in the ECM are correlated with alterations in ECM stiffness and adhesion forces exerted by the ECM during aging (Figure 3). Most importantly those changes are strongly dependent on the level of lung stretch. In this regard, it is noticeable that ECM during aging is softer when measured at RV but it becomes stiffer when measured at FV. A similar response was observed when unspecific adhesion forces were measured, being higher only in lung decellularized samples from young animals in RV. However, no changes in viscoelasticity were detected. Our findings may be related to the organization of collagen bundles at the microscale, as it has been reported that older collagen fibers display a straighter appearance and are less prone to waviness. In this connection, these differences in straightness or waviness are likely related to differences in collagen fibrils crosslinking at the nanoscale (Sobin et al., 1988). A such, we speculate that collagen in older lungs may be crosslinked in a way to make it organize in a straighter manner, giving rise to a more compliant response in unstretched conditions (RV), but that such organization may lead to a strong strain-hardening response under stretched (FV) conditions. Conversely, the crosslinking of collagen fibrils in younger lungs may be more isotropic, giving rise to a less compliant unstretched lung but also less prone to strain-hardening. While these are interesting speculations, a nanoscale analysis of collagen fibrils and their crosslinks would be needed to confirm them, followed potentially by computational analysis to assess what would be the response to different stretch (thus inflation) levels.

As proof of concept, we employed these lung scaffolds from young and aged animals and at RV and FV conditions to repopulate MSCs. Despite the distinct biomechanical characteristics, we observed comparable cell attachment, viability, and proliferation across all conditions (Figure 4) in concordance with Sokocevika and Wagner et al. findings on aged lungs (Sokocevica et al., 2011; Wagner et al., 2014). These results suggest that the alterations in ECM composition and its mechanical properties are not enough to modulate MSCs proliferation as occurs in other diseases such pulmonary fibrosis with more relevant biomechanical alterations (Júnior et al., 2021; Qin et al., 2023). However, this model holds promise for advancing our comprehension of how aging and tissue stretch may alter other biological aspects which could be more susceptible such as cell differentiation and motility in MSCs and other pulmonary cells. Consequently, this work opens the opportunity to better understand the association of aging with regenerative medicine, lung cancer, and other chronic pulmonary diseases in more specifically designed studies.

## 5 Conclusion

This work provides novel insights into pulmonary ECM biomechanical alterations associated with aging at non-inflated (RV) and physiologically inflated (FV) lungs. Our findings indicate that aging induces changes in ECM protein composition, leading to ECM stiffness and adhesion force modifications depending on lung volume (RV and FV). The ECM biomechanical alterations caused by aging did not modify MSCs viability and proliferation. However, the use of decellularized lung scaffolds from aged and/or other lung disease murine/human under physiomimetic conditions (FV) is crucial for a comprehensive understanding of how biomechanical properties in the lung may contribute to susceptibility and progression of chronic lung diseases, lung development and cancer.

## Data Availability

The raw data supporting the conclusion of this article will be made available by the authors, without undue reservation.
